# An Unprecedented Aggregation of Whale Sharks, *Rhincodon typus*, in Mexican Coastal Waters of the Caribbean Sea

**DOI:** 10.1371/journal.pone.0018994

**Published:** 2011-04-29

**Authors:** Rafael de la Parra Venegas, Robert Hueter, Jaime González Cano, John Tyminski, José Gregorio Remolina, Mike Maslanka, Andrea Ormos, Lee Weigt, Bruce Carlson, Alistair Dove

**Affiliations:** 1 Proyecto Dominó, Comisión Nacional de Áreas Naturales Protegidas, Cancún, Quintana Roo, México; 2 Center for Shark Research, Mote Marine Laboratory, Sarasota, Florida, United States of America; 3 Smithsonian Conservation Biology Institute, National Zoological Park, Washington, D.C., United States of America; 4 Laboratories of Analytical Biology, National Museum of Natural History, Smithsonian Institution, Washington, D.C., United States of America; 5 Georgia Aquarium Research Center, Georgia Aquarium, Atlanta, Georgia, United States of America; Institute of Marine Research, Norway

## Abstract

Whale sharks, *Rhincodon typus*, are often perceived as solitary behemoths that live and feed in the open ocean. To the contrary, evidence is accumulating that they are gregarious and form seasonal aggregations in some coastal waters. One such aggregation occurs annually north of Cabo Catoche, off Isla Holbox on the Yucatán Peninsula of Mexico. Here we report a second, much denser aggregation of whale sharks (dubbed “the Afuera”) that occurs east of the tip of the Yucatán Peninsula in the Caribbean Sea. The 2009 Afuera event comprised the largest aggregation of whale sharks ever reported, with up to 420 whale sharks observed in a single aerial survey, all gathered in an elliptical patch of ocean approximately 18 km^2^. Plankton studies indicated that the sharks were feeding on dense homogenous patches of fish eggs, which DNA barcoding analysis identified as belonging to little tunny, *Euthynnus alletteratus*. This contrasts with the annual Cabo Catoche aggregation nearby, where prey consists mostly of copepods and sergestid shrimp. Increased sightings at the Afuera coincide with decreased sightings at Cabo Catoche, and both groups have the same sex ratio, implying that the same animals are likely involved in both aggregations; tagging data support this idea. With two whale shark aggregation areas, high coastal productivity and a previously-unknown scombrid spawning ground, the northeastern Yucatán marine region is a critical habitat that deserves more concerted conservation efforts.

## Introduction

The whale shark, *Rhincodon typus*, is a planktivorous, filter-feeding elasmobranch that lives in tropical and subtropical oceans throughout the world and is the longest and heaviest of all fishes [1]. The International Union for the Conservation of Nature lists the whale shark as “Vulnerable” in the 2010 Red List of Threatened Species [2]. Population genetic structure has been investigated and some estimates of effective population size have been made [3], but the actual number of whale sharks inhabiting the world's oceans is unknown.

Aggregations of whale sharks have been reported from at least eight tropical locations around the world [4], [5], [6]. These aggregations range from a few individuals to a few dozen and all are associated with locally high concentrations of zooplankton. This paper describes the recent discovery of an enormous aggregation of whale sharks, the largest ever reported, off the Yucatán peninsula of Mexico. This spectacular biological phenomenon provides an opportunity to monitor regional populations and also delineates a previously unreported scombrid spawning area.

Mexican fishermen from the villages of Holbox and Chiquilá, located on the northeastern coast of Quintana Roo on the Yucatán Peninsula, first reported summer sightings of whale sharks to author RH in 2002. Fishermen were apparently aware of the presence of whale sharks in adjacent waters for many years, perhaps generations, but did not harvest them and did not bring their observations to the attention of researchers. The revelation of substantial numbers of whale sharks in Quintana Roo coastal waters prompted the Mexican federal natural resources agency CONANP to establish the Domino Project in 2003. This multi-institutional research and conservation program was aimed at investigating different aspects of whale shark biology and understanding the importance of the Quintana Roo aggregation, in partnership with the growing whale shark ecotourism industry. Surveys of the Holbox aggregation by boat began in 2003 and aerial surveys began in 2005. Together, these approaches were used to document the size of the local population, the size of individual sharks and the sex ratio in the area where whale sharks were observed.

Whale sharks gather in coastal waters between Cabo Catoche and Isla Contoy on the northeastern tip of the Yucatán Peninsula, beginning in May and dispersing in mid-September, with peak abundance varying between late July and mid August [7], [8]. Transient individuals may also be observed in the area during April, October, and other months, but the vast majority of sharks is present from May to September.

Since 2003, “whale shark watching” businesses have transformed the village of Holbox from a fishing-based economy to an ecotourism destination and these businesses have also proliferated on Isla Mujeres and in Cancún. As a step towards improved conservation and better management of whale sharks as a sustainable resource, the Mexican government in June 2009 established Reserva de la Biosfera Tiburón Ballena, or the Whale Shark Biosphere Reserve, adjacent to the existing natural reserve of Yum Balam (Official decree available at: http://www.conanp.gob.mx/sig/decretos/reservas/Tiburon.pdf). The biosphere reserve was designated to include all of the primary locations where whale sharks had been reported between Holbox and the northern tip of Isla Contoy.

This study was prompted by reports of a second and apparently quite different whale shark aggregation occurring outside the Cabo Catoche area and further offshore.

## Results

**Figure 1:**
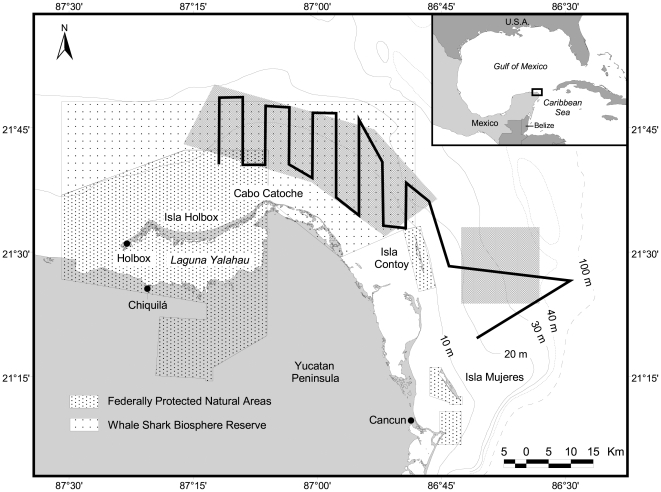
The flight path followed on each aerial survey for whale sharks off the coast of Quintana Roo, México. The triangular leg to the east of 86°45′W was added to the original survey design to incorporate the newly-discovered Afuera whale shark aggregagtion. Waypoints were marked on GPS instrumentation to ensure accurate repeatability of the same path.

In September 2006, author RPV observed a second, more remote aggregation of whale sharks farther to the southeast (hereafter referred to as the Afuera or “outside” aggregation) in the offshore waters between the latitudes of Isla Contoy and Isla Mujeres. These observations confirmed anecdotal information provided by fishermen of an offshore aggregation from as early as 1991. Five aerial surveys were made over the area (Figure 1) in September 2006, during which a total of 480 sightings was made. On an aerial survey on September 7^th^, author JFGR counted 207 whale sharks at the Afuera location, and one photograph taken on September 10th showed 76 whale sharks apparently feeding at the surface in blue water. This was surprising at the time because all of the work near Cabo Catoche had shown that the whale sharks were feeding in dense patches of crustacean zooplankton in somewhat more turbid, green and shallow water (6–20 m deep) close to shore. We hypothesized that sharks in the Afuera aggregation were most likely feeding on fish eggs, which are mostly transparent and can occur in relatively high abundance without greatly affecting water clarity. We also surmised that the density of eggs had to be very high given the number of sharks feeding in the area and the abundance of plankton at the alternative feeding site near Cabo Catoche.

**Figure 2:**
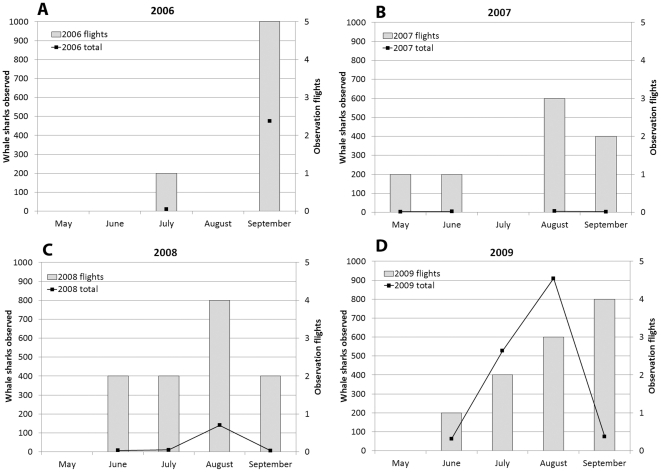
Aerial survey effort and whale sharks observed per month between May and September, from 2006 to 2009, in the coastal waters of Quintana Roo, México.

**Figure 3:**
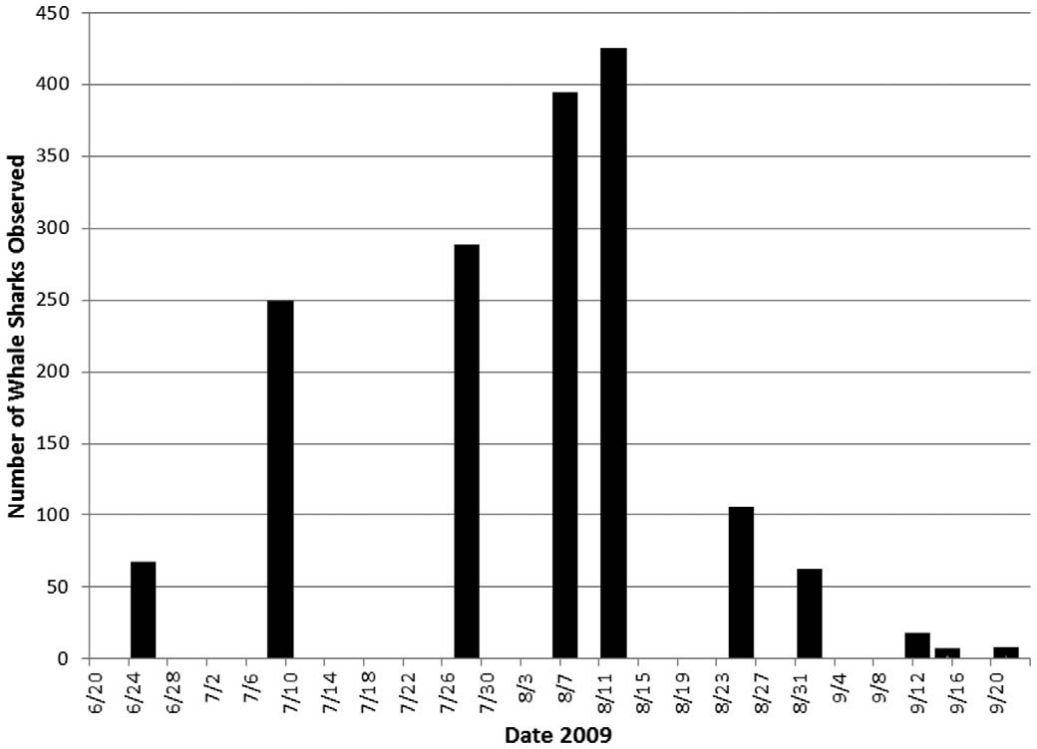
Time series of whale shark observations during the 2009 Afuera whale shark aggregation in the coastal waters of Quintana Roo, México. Each column represents a single aerial survey.

**Figure 4:**
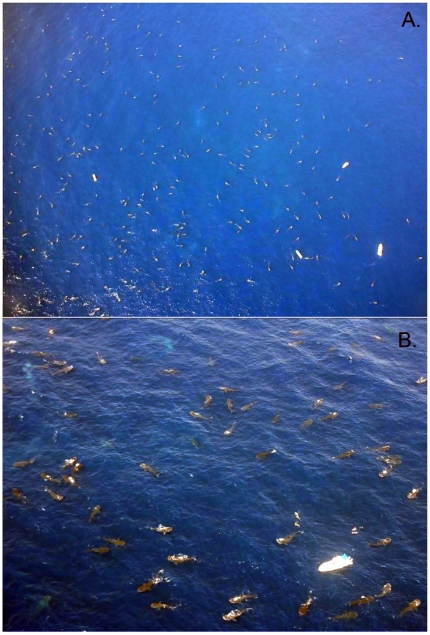
Aerial photographs of whale sharks feeding at the Afuera aggregation in August 2009. *Figure 4A* was taken from approximately 600 m altitude and shows 220 whale sharks and 4 tourist boats. *Figure 4A* was taken from lower altitude and shows 68 whale sharks, 1 tourist boats and 2 pairs of tourists snorkeling.

In 2007 the Afuera aggregation either failed to materialize or, less likely, was missed by observers, despite seven survey flights over the period from May to September (Figure 2B). Whale sharks were recorded more consistently at the Afuera site during 2008, including 87 animals on one flight in August (Figure 2C). In 2009, however, animals were noticed on the Afuera site earlier in the year (May) than in the previous years, and in steadily increasing numbers; during June and July it became clear that totals in 2009 would be much higher (Figure 2D and Figure 3). The concentration of activity at the Afuera site peaked on August 8th, 2009 when 389 animals were seen on a single flight, and on August 12th, 2009, when 420 animals were recorded (Figure 4). These gatherings occurred in elliptical areas of ocean approximately 3 km by 6 km.

**Figure 5:**
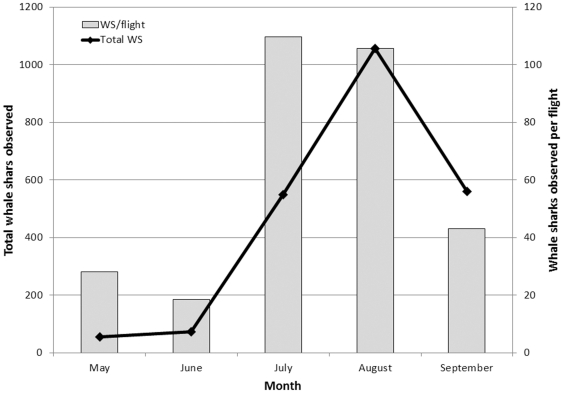
Total number or whale sharks and whale sharks per flight, compiled from aerial surveys of the Afuera whale shark aggregation off the coast of Quintana Roo, México between 2005 and 2009.

From 2005 to 2009, a total of 2,295 whale shark sightings was recorded on 34 flights over the Afuera area (Figure 5) at an average of 67.5 whale sharks per flight. While the total number of sightings recorded certainly includes many repeated sightings of the same animals, the sightings on any given day represent unique animals (see methodology for details), so we can be confident that the Afuera aggregation involved at least 420 animals, making it the largest whale shark aggregation ever recorded, by far. Sightings in 2009 totaled an order of magnitude more than the Afuera the previous year, and many more animals than are usually seen at the more consistent Cabo Catoche aggregation.

A total of 81 whale sharks were tagged at the Afuera in 2009, using conventional visible numbered tags. Of these, only one was re-sighted at the Cabo Catoche aggregation area during the same year. Conversely, three whale sharks were tagged at the Cabo Catoche area in 2009 and none of these was re-sighted at the Afuera aggregation. Fourteen animals that had been tagged at Cabo Catoche in previous years were re-sighted in 2009; all of these were re-sighted at the Afuera, while only one was seen at Cabo Catoche. Of the animals tagged at the Afuera, 57 were male and 20 were female, while four were of undetermined gender. This male: female ratio of 2.85∶1 at the Afuera is similar to the ratio at Cabo Catoche, where it averaged 2.64∶1 for the period 2003–2009.

Plankton collected in 2008 at the Cabo Catoche feeding site consisted of mixed crustacean zooplankton, with sergestid shrimp (*Lucifer faxoni*) and calanoid copepods occurring in higher abundance within feeding areas than in adjacent areas where whale sharks were not feeding [9]. Plankton collected at the Afuera site in 2009 consisted almost entirely of fish eggs. The abundance of eggs was so high that the tow duration was reduced to just 40 seconds so as not to clog the net. Nutritional analyses showed that the mixed zooplankton from Cabo Catoche and the fish eggs from the Afuera were surprisingly comparable in energy density and basic nutritional composition. Mixed zooplankton had slightly higher energy density (0.39 Kcal/g) than fish eggs (0.30 Kcal/g). Crude calculations of energy intake based on prey density and standardized time spent feeding, however, differed markedly between the two sites; 8 hrs feeding at Cabo Catoche (7.1 g/m^3^) might yield around 11,000 Kcal, whereas the same time spent at the Afuera (21.1 g/m^3^) might yield around 27,000 Kcal for an average-sized whale shark [9].

Fish eggs from the Afuera plankton samples were subjected to *Cox1* DNA barcoding. There were 6 haplotypes identified (GenBank accession numbers HM586985–HM586990), with no sequence differing by more than 2 bases from the common haplotype, which was:

CCTTTATCTAGTATTCGGTGCATGAGCTGGTATAGTTGGCACGGCCTTAAGCTTGCTCATCCGAGCTGAACTAAGCCAACCAGGTGCCCTTCTTGGGGACGACCAGATCTACAATGTAATCGTTACGGCCCATGCCTTCGTAATGATTTTCTTTATAGTAATGCCAATTATGATTGGAGGGTTTGGAAACTGACTCATCCCTCTTATGATCGGAGCTCCAGACATAGCATTCCCTCGAATAAATAACATGAGCTTCTGACTTCTTCCCCCATCTTTCCTTCTACTCCTAGCTTCTTCAGGAGTTGAGGCCGGTGCCGGAACTGGTTGAACAGTCTACCCTCCGCTTGCCGGAAATCTGGCCCATGCCGGAGCATCCGTTGACTTAACCATTTTCTCCCTCCATCTAGCAGGTGTTTCCTCAATTCTTGGGGCAATTAACTTCATTACGACAATTATCAACATGAAGCCTGCCGCTATTTCTCAGTATCAAACCCCTCTATTCGTATGAGCTGTACTAATTACGGCCGTTCTTCTTCTGCTATCCCTCCCAGTCCTTGCCGCTGGCATTACAATGCTCCTGACAGACCGAAACTTAAATACAACCTTCTTCGACCCTGCAGGCGGGGGAGATCCAATCCTTTACCAACACCTATTC

This sequence provided a clear identity match with little tunny, *Euthynnus alletteratus*. This small to medium scombrid was not previously known to spawn off the Yucatán, but the time of year, duration of spawning and prevailing conditions are consistent with other little tunny spawning grounds in the Mediterranean Sea [10]. It is not clear whether additional pelagic species may also have participated in this spawning event.

## Discussion

The ocean surrounding the northeast coast of the Yucatán Peninsula is a rich area for billfishes (*Makaira* and *Istiophorus* spp.), common dolphin fish (*Coryphaena hippurus*), tunas (*Thunnus* spp.), groupers (*Epinephelus* spp.) and snappers (*Lutjanus* spp.). Only more recently has it also become known to scientists as an aggregation site for whale sharks, manta rays, devil rays, cownose rays, and sea turtles. The biological richness of this area is likely to be associated with tropical upwelling that brings nutrients onto the Yucatán shelf from deeper water to the southeast, resulting in higher productivity than might otherwise occur in inshore tropical waters [11], [12], [13].

The observations of whale sharks farther offshore was initially surprising, because the blue water there suggested low concentrations of plankton and a less productive food source than at the green water site at Cabo Catoche. We hypothesized that the whale sharks at the Afuera were feeding on an alternate food source, most likely fish eggs. Whale sharks are known to aggregate at Ningaloo Reef in Australia, where corals are spawning [14], but coral spawn produces obvious, milky slicks on the surface. By contrast, pelagic fish eggs are usually transparent and therefore a more likely food source for the whale sharks at the Afuera. A similar but much smaller aggregation of whale sharks reported from Gladden Spit, Belize, was related to mass spawning of snappers [15], [16], whereas in the north-central Gulf of Mexico, a smaller aggregation of *R. typus* was associated with a fish spawning event [4]. In the latter study, the primary egg morph at the aggregation site was verified by genetic analysis as little tunny, *Euthynnus alletteratus*. Plankton sampling at the Afuera confirmed our hypothesis that fish eggs were the primary food item and DNA barcoding also identified the little tunny, *E. alletteratus*, as the main species involved, although it remains a possibility that other scombrid species are also involved, since these species are known to form multi-species spawning assemblages [17], [18]. Nothing is known currently about the size of the little tunny population or precisely where and when they spawn. Clearly the northeast Yucatán group must comprise a large number of fish, considering the size of the Afuera area, the abundance of eggs obtained from plankton tows, the persistence of the aggregation from May to September and the large number of whale sharks feeding on the eggs at any one time.

The higher number of whale sharks observed at the Afuera in 2009 than previous years may reflect an exceptional year for little tunny spawning, or that more whale sharks were attracted to the Afuera and away from other sites than in previous years. It is also possible that quantitative or qualitative changes in plankton composition at the Cabo Catoche site caused the animals to seek different feeding grounds. The data certainly do not support increased surveillance alone as an explanation for the 2009 event. The number of flights was comparable between 2008 and 2009, but many more animals were observed in 2009 (Figures 2 and 3).

The increased number of animals recorded at the Afuera in 2009 coincided with a marked drop in animals observed at the Cabo Catoche site, where we had previously recorded up to 145 animals. In addition, the sex ratio of around 2.8 males to 1 female was similar at the Afuera to the historical average at Cabo Catoche (2.6∶1). Taken together, these results support the idea that the Afuera animals simply relocated from Cabo Catoche in 2009. Within 2009, however, there seems to have been little movement between the two sites; of the 81 animals tagged at the Afuera, many were re-sighted at the Afuera and only one was re-sighted at Cabo Catoche, whereas of the three animals tagged at Cabo Catoche, none was re-sighted at the Afuera.

By number of animals, the Yucatán Peninsula is arguably the largest and most important known aggregation area for whale sharks anywhere in the world. At least two aggregation sites are present in this region: the green water site north of Cabo Catoche and the blue water Afuera location reported herein. In addition, this study has shown indirect evidence that a significant and persistent scombrid spawning event also occurs at the Afuera site; little tunny were not previously known to spawn in this area. The large numbers of manta rays (*Manta* spp.), devil rays (*Mobula* spp.), cownose rays (*Rhinoptera bonasus*) and sea turtles observed during the aerial surveys suggest that this is a highly productive and diverse marine ecosystem. For all of these reasons, the marine realm of the northeast Yucatán should be considered a hotspot of marine biodiversity and a priority region for *in situ* conservation efforts. The proximity of the area to a major tourist destination (Cancún, Isla Mujeres and the Riviera Maya) places the area at an additional risk of negative impacts from human activities. Extraordinary biological phenomena of the sort we report here deserve extraordinary conservation measures.

## Materials and Methods

Research for this publication was carried out with prior permission of the Mexican federal government agency CONANP and was reviewed and approved by the conservation, research and animal care committee at Georgia Aquarium.

A systematic aerial survey for whale sharks in the marine waters off the northeastern Yucatán Peninsula was designed by author RP based on prior experience with marine mammal, crocodile and shark surveys since 1983. The design was fundamentally similar to that described in Rowat et al. [19]. Briefly, it involved flights departing from Cancun airport General Aviation terminal in a Cessna 206 aircraft and then flying a zig-zag sequence of parallel paths between fixed GPS waypoints at an altitude of 500 m, designed to provide complete observational coverage of the area of interest. During 2004 and 2005, five observers and two camera people were trained and calibrated for aerial observations of whale sharks using this approach. Of this team, two observers, one camera person and author RP were present on every flight. A 500 m observation distance on either side of the aircraft was achieved by having observers look in the area from 45 degrees down towards the plane, and also marked using tape on the windows for reference; these approximations were calibrated against markings on the ground using a marked football field in Cancun as a guide.

A total of 61 flights was carried out, totaling over 105 hours of transect time; 34 flights included the Afuera aggregation area after it was discovered in 2006. Transects lasted and average 1.25 hrs with a minimum annual average in 2006 (57 min) and a maximum annual average in 2009 (1 hr, 27 min). All surveys were conducted in the morning, around 0930 hrs, because past experience had shown that whale sharks tend to stop feeding and submerge around mid-day, thereby becoming less visible from the air. Regardless of the area studied, the main feeding period for the whale sharks was between 0830 and 1130 hrs and always occurred at the surface. The exclusively surface-feeding behavior of whale sharks in the Yucatan has been repeatedly confirmed by snorkeling and SCUBA diving at both the Cabo Catoche and Afuera locations. Two spotters accompanied the pilot to record the number of whale sharks observed, to take aerial photographs, and to mark the latitude and longitude of each sighting with a GPS data-logger. In addition, a camera person was seated in a safety harness on the right side of the aircraft and the door removed, so that this person could make observations and take photographs directly beneath the aircraft. This configuration allowed observation of up to 500 m on either side of the aircraft and the “blind spot” directly beneath.

Each flight followed the path shown in Figure 1 and was conducted at an altitude of 500 m and a ground speed of 95 km/h. Wind speed and direction, sea conditions, water color, cloud coverage and average temperature were recorded. Wind speeds over 40 km/h, or above 5 on Beaufort scale, resulted in poor sightings, so surveys were avoided on such days. The pre-determined flight path was never interrupted, except over the afuera aggregation, which was so compact and replete with animals that it was necessary to make several tight circles with the aircraft in order to get accurate counts. On these occasions, after gaining several replicate counts at 500 m, the altitude was increased to 1500 m to get a view of the whole aggregation and to collect a photographic mosaic which could be used later to confirm the count. For all counts, both main observers made counts of animals on their side of the aircraft using manual digit counters (counting clickers) and then after the flight the numbers were added together to obtain the total count.

Author RPV used his personal boat to locate the Afuera aggregation using GPS coordinates radioed from the aerial survey group. Upon reaching the whale sharks, a record was made of the general behavior of the animals (e.g. “feeding” or “not feeding”) and then sharks were selected haphazardly for tagging. Stainless steel-headed dart tags with plastic-coated stainless steel leaders were attached to a bright yellow, hard plastic numbered placard 10 cm×20 cm (similar to that shown in Graham and Roberts, [20]) and applied to the sharks in the dorsal musculature, left-lateral to the first dorsal fin, using a pole spear. The size of each tagged whale shark was estimated to the nearest half-meter by positioning the research boat parallel and as close as possible to each shark and then measuring against a metric scale marked on the side of the boat.

A total of 152 surface trips was made, incorporating over 760 hrs of observation time. On each occasion, observations were made of water conditions (depth, temperature, pH secchi disk), weather, and whale shark factors (number, size, sex, behavior, wounds).

Plankton tows were conducted inside and immediately outside of the feeding aggregations at both the Cabo Catoche and Afuera locations. A 200-micron mesh, square framed neuston net was used [9]. Tow durations were standardized at two minutes at Cabo Catoche, but at the Afuera this tow duration resulted in the net becoming clogged, so the sampling time was adjusted to only 40 seconds. Aliquots of the plankton collected in this fashion were preserved immediately in 10% formalin for morphological vouchers, and frozen for DNA and nutritional analysis. Energy density was determined from nutrient composition: crude protein (CP) was determined by Kjeldahl method, fat (F) by acid hydrolysis and ash (A) by dry oxidation, carbohydrate (CHO) by difference, and then caloric density calculated according to the equation:
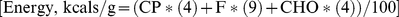
Fish eggs were identified to species using *Cox1* mitochondrial DNA barcoding [21]. Genomic DNA was extracted from egg samples (4 parallel of 12–15 eggs, 3–5 eggs and 1 egg) via an automated phenol-chloroform DNA extraction on the Autogenprep965 (Autogen, Holliston, MA) using the mouse-tail tissue protocol with a final elution volume of 100 µl. For PCR, 1 µl of this genomic DNA is used in a 10 µl reaction with 0.1 µl Bioline (BioLine USA, Boston, MA) taq polymerase according to manufacturer's protocol. Fish barcode primers used were: FISH-BCL 5′-TCAACYAATCAYAAAGATATYGGCAC and FISH-BCH 5′-TAAACTTCAGGGTGACCAAAAAATCA Baldwin et al. [22].

Thermal cycler program for PCR was one cycle of 5 m@95°C; 35 cycles of 30 s@95°C, 30 s@52°C and 45 s@72°C; one cycle of 5 m@72°C, and a hold at 10°C. Additionally, fish eggs (3 parallel of 12–15, 3, and single egg) were smashed onto FTA cards (Whatman). After two weeks of storage they were punched, washed and dried following the Whatman protocol for treatment prior to PCR amplification. Ten parallel punches were processed for single egg, and two punches for the multiple egg FTAs. Primers and cycling conditions were same as above.

PCR products were purified with Exosap-IT (USB, Cleveland, OH) using 2 µl of 0.2× enzyme and incubating for 30 m@37°C then inactivating the reaction for 20 m@80°C. Sequencing reactions were performed using 1 µl of this purified PCR product in a 10 µl reaction containing 0.5 µl primer, 1.75 µl BigDye buffer and 0.5 µl BigDye (ABI, Foster City, CA) and run in the thermal cycler for 30 cycles of 30 s@95°C, 30 s@50°C, 4 m@60°C and then held at 10°C. These sequencing reactions were purified using Millipore Sephadex plates (MAHVN-4550; Millipore, Billerica, MA) per manufacturer's instructions and stored dry until analyzed. Sequencing reactions were analyzed on an ABI 3730XL automated DNA sequencer and sequence trace files were exported into Sequencher 4.7 (GeneCodes, Ann Arbor, MI). Sequence ends were trimmed until the first and last 10 bases contained fewer than 5 base calls with a confidence score (phred score) lower than 30. After trimming, forward and reverse sequences for each specimen were assembled, each assembled contig was examined and edited by hand, and each sequence was checked for stop codons. Finally the consensus sequence from each contig was aligned and exported in text format. Sequences were compared to the Smithsonian's reference fish database for species identifications, and were submitted to GenBank with accession numbers HM586985–HM586990.
